# A systematic review of magnetic versus conventional ureteric stents for short term ureteric stenting

**DOI:** 10.1007/s11845-022-02920-3

**Published:** 2022-01-22

**Authors:** Louise Lyons, Ned Kinnear, Derek Hennessey

**Affiliations:** 1 Department of Urology, Mercy University Hospital, Cork, Ireland; 2 Adelaide Medical School, University of Adelaide, Adelaide, Australia

**Keywords:** Magnetic stent, Pain, Ureteric stent

## Abstract

Ureteric stents play an essential role in urology. However, patients can suffer a range of stent-related symptoms with stent in situ and during removal. Conventional ureteric stents are removed using a flexible cystoscopy, whereas magnetic stents may be rapidly removed with a smaller catheter-like retrieval device. The primary aim of this systematic review was to compare the morbidity including pain associated with conventional versus magnetic ureteric stents. The secondary aim was cost comparison. Searches were performed across databases, including Medline, Scopus, Embase and Cochrane. This review was performed in accordance with Preferred Reporting Items for Systematic Reviews and Meta-Analyses (PRISMA). The search from the 5 databases returned a total of 358 articles. After duplicates were removed as well as the inclusion and exclusion criteria applied, a total of 6 studies were included in the final review. Ureteric Stent Symptoms Questionnaire (USSQ) and Visual Analogue Score (VAS) were used in most of the studies. All the studies reported that magnetic ureteric stents resulted in a reduction in the pain on the removal of magnetic ureteric stents, and no statistically significant difference with indwelling ureteric stents. Furthermore, majority of the studies reported a reduction in the cost associated with magnetic ureteric stents. There is no significant difference in pain from indwelling ureteric stents. There is a reduction in pain with the removal of magnetic ureteric stents compared to conventional removal via cystoscopy and an associated reduction in cost.

## Introduction

Ureteric stents are used to maintain ureteric patency in the face of post-instrumentation oedema, to bypass a stricture and facilitate drainage while a ureteric injury or anastomosis heals. Modern ‘double J’ (JJ) stents are named for their pliable ‘J’ coil at either end, which reduces stent migration [1, 2]. Since their introduction, multiple materials have been trialled, with silicone and polyurethane among the most frequently used [3].

Ureteric stents can cause a range of symptoms, including frequency, urgency, haematuria, incomplete emptying as well as flank and suprapubic pain [4–6]. In addition, ureteric stent removal by flexible cystoscopy can be uncomfortable and is associated with additional health care costs [1, 7]. Ureteric stent symptoms can have a significant impact on patients’ quality of life. Subsequently, developments in stent design have focused on a different mechanism of removal, such as stents tipped with strings or a magnet [1, 7]. The use of magnetic ureteric stent was first reported in 1989. However, their uptake has been slow due to unfamiliarity and unclear efficacy [8].

The primary aim of this study was to compare the morbidity associated with magnetic versus conventional ureteric stents both in situ and at stent removal. The secondary aim was the cost-effectiveness of each stent type.

## Methods

**Fig. 1:**
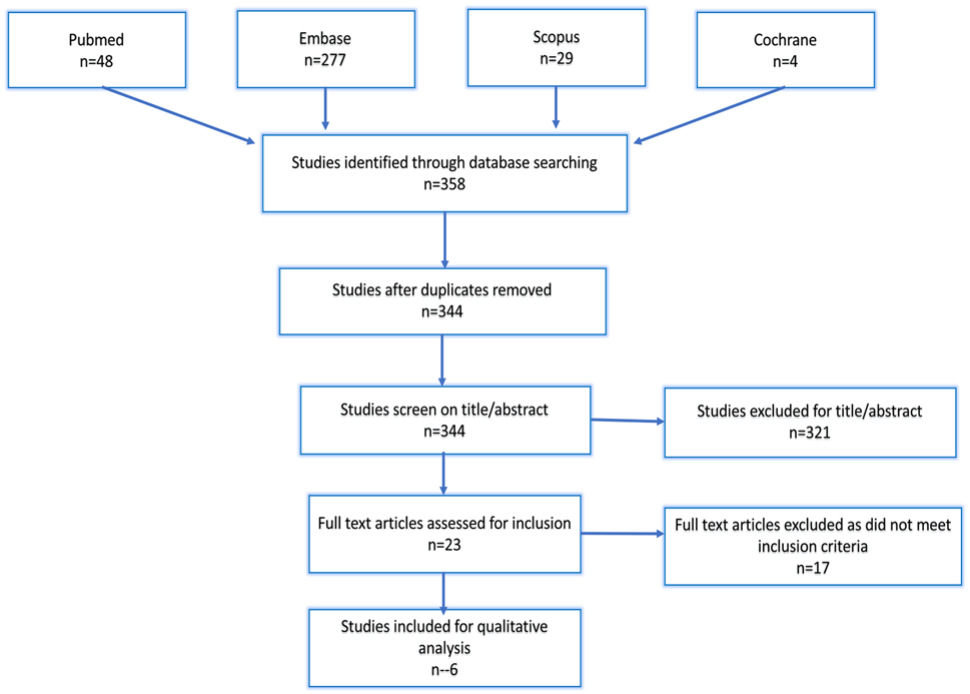
PRISMA flow diagram

The review was registered with the International Prospective Register of systematic reviews (PROSPERO) (registration number CRD42021234021), and the protocol is available online [9]. Searches were performed by title and abstracts in medical databases Pubmed, Embase, Scopus and Cochrane using search terms, “ureteric stent”, “magnetic”, “pain” using the Boolean operator (AND). The first search date was September 13, 2020, and a final search was March 3, 2021. Two authors (LL and DH) independently screened results by title and abstract to select articles for full-text review. Eligible articles then progressed to data extraction, performed independently by LL and DH using a pre-defined form. Disagreements were resolved by discussion. The final list of included articles was determined by the consensus of all the authors. An additional secondary search of the bibliography of all selected papers was performed. Grey literature was eligible, including conference proceeding and internet articles, if these met the inclusion criteria below. For identified potentially eligible works, significant correspondence was attempted with study authors to resolve unclear raw outcome data instances. Our method for identifying and evaluating data complied with the Preferred Reporting Items for Systematic Reviews and Meta-analyses criteria [10] (Fig. 1).

### Inclusion criteria

Inclusion criteria were determined utilising the patient population, intervention, comparator, outcome and study (PICOS) method [11]. Eligible studies included only patients with ureteric stents (P), presented groups with magnetic (I) and conventional stents (C), assessed pain or cost (O) and were comparative in nature (S). Eligible studies were original, written in English, published after 01/01/1989 to 30/11/2020 and available in full text.

### Exclusion criteria

Exclusion criteria lacked original raw data, had a significant overlap with a larger included study, animal-based.

### Intended analysis

The primary outcomes were patients’ reported morbidity. The secondary outcome was the cost. A qualitative summary was intended for all the data. Quantitative analysis was intended if studies with sufficiently homogenous methodology were identified. All the analyses were two-tailed, and significance was assessed at the 5% alpha level.

### Bias

The authors expected to identify few randomised controlled trials if any. Subsequently, the risk of bias for (comparative) cohort studies was assessed utilising the Newcastle Ottawa Scale, as prescribed by the Cochrane Handbook [12]. For non-comparative case series, study quality was measured with the modified Delphi checklist, as recommended by a recent systematic review of quality assessment tools [13]. Given the expected nature of included studies, three of nine items on the Newcastle Ottawa Scale were inapplicable and not scored, as were three of eighteen modified Delphi criteria. Study quality was independently assessed by two reviewers (LL and DH) against pre-defined criteria. Disagreements were resolved by discussion. The risk of bias was not used to exclude studies. We anticipated identifying too few studies to assess publication bias.

## Results

### Study design

The results of the search are summarised in Fig. 1. Three hundred and fifty-eight results were identified across four databases. After the removal of fourteen duplicates, a title and abstract review was performed, with 23 articles proceeding to the full-text review. Six studies were eligible for inclusion (Fig. 1) comprising 1 randomised control trial, 3 observational case–control studies and 2 cohort studies (Table 1). Studies were inadequately homogenous to perform a meta-analysis of any outcomes.

**Table 1:** Eligible studies

**Year, First author**	Study design	Duration of stenting	Patient number	Pain with stent in situ (USSQ Questionnaire)	Pain of stent removal VAS (0–10)	Cost analysis
**2019 Pohlman**	Cohort Study Non-comparative	Only magnetic stent used - removed 10 to 12 days after transplantation	7	Magnetic stent: 33% - bladder area 16.7% - flank 16.7% - penis	Magnetic stent (2.6)	Saved €130 euro per patient with magnetic stent
**2019 O’Kelly**	Observational Case control	Magnetic - 5.5 days Conventional - 21.5 days	100	No differentiation	Magnetic stent (2.9) Conventional stent (3.9)	Saved €203 per patient with magnetic stent
**2017 Seveenco**	Observational Case control	Ureterorenoscopy - 1 week for both magnetic and conventional stents Laparoscopic pyeloplasty - 4 weeks for both magnetic and conventional stents	151	No differentiation	Magnetic stent (2.5) Conventional stent (6.0)	Saved €203 per patient with magnetic stent
**2017 Rassweiler**	Randomised control trial	Both type of stents removed after 7 to 14 days	40	Magnetic stent: 48% - lower abdomen/bladder area 18% - flank 3.7% - penis Conventional stent: 54% - flank 14.29% - groin/penis	Magnetic stent (3) Conventional stent (4)	Reduction in cost of €101.41 using the magnetic stent
**2019 Capocasale**	Observational Case control	Magnetic stent removed after 4 weeks	100	93% - comfortable 7% - distressed	Decreased pain on removal of magnetic stents	No cost analysis
**2018 O’Connell**	Retrospective cohort study Non-comparative	Magnetic stent removed after 5.8 days	59	25% - debilitating pain	90.7% reported satisfaction or very satisfied	Potentially saving €47,790 over the 9-month period

### Primary outcomes

All the studies included in this review assessed for differences in stent-related symptoms between conventional stents and magnetic stents. Rassweiler et al. performed a randomised control trial to assess the impact of magnetic ureteric stents on patient’s quality of life and discomfort during the removal [14]. Forty patients that required a ureteric stent following a ureteroscopy were randomised prospectively. This study used the USSQ to assess the symptoms of the indwelling ureteric stents and the VAS for their removal. The ureteric stents were removed between 7 and 14 days after surgery. To prevent skewing of the results, the USSQ was completed 5 days post-ureteroscopy to avoid any influence on the procedure itself. The VAS scores showed the magnetic ureteric stent with a mean of 3 and the conventional ureteric stent with a mean of 5. Magnetic ureteric stents were removed in a shorter time frame of 9.55 min compared to the flexible cystoscopy used in conventional ureteric stents, which was 21.35 min. In addition, this study had a failure rate for removal of magnetic ureteric stent of 2%, necessitating flexible cystoscopic removal [14].

Capocasale et al., O’Kelly et al. and Sevcenco et al. are all observational case–control studies [15–17]. O’Kelly et al. and Sevcenco et al. compared magnetic with conventional ureteric stents regarding morbidity, pain on removal complications and cost effectiveness [16, 17]. In contrast, Capocasale et al. report on the morbidity, outcomes and safety relating to the use of magnetic ureteric stents in 100 patients who have undergone a kidney transplant [15]. O’Kelly et al. is a comparative study performed across two different sites, with 50 patients in each arm, all of whom underwent ureteroscopy [16]. This study found no significant difference between magnetic and conventional ureteric stents USSQ score 14.3 vs. 15.3, *p* = 0.32 [16]. Site A (magnetic) had their participants complete the USSQ at the time of stent insertion, whereas Site B (conventional) had their patients complete the USSQ within 5 months via the postal service [16]. Sevcenco et al. performed a comparative observational study of 151 patients, 12 of whom had laparoscopic pyeloplasty, the remainder went ureteroscopy for urinary calculi [17]. Magnetic stents were inserted in 118 patients and conventional ureteric stents in 33 patients [17]. Sevcenco et al. was the only study to report a significant difference between the two types of stents for indwelling pain [17]. However, they did not use the validated USSQ but an adapted VAS for indwelling stent irritation (VAS 1) [17]. Stent irritation was marginally higher in the magnetic ureteric stent than the conventional ureteric stent with a VAS 1 score of 3 and 2, respectively [17].

The two observational case–control studies showed a significant reduction of pain on the removal of the magnetic stents compared to conventional ureteric stents [16, 17]. O’Kelly et al. reported pain on the removal of magnetic ureteric stent and conventional ureteric stent with VAS scores of 2.9 and 3.9, respectively [16]. Sevcenco et al. reported a more significant difference between the two groups with a VAS score of 2.5 for magnetic stent removal compared to a score of 6 for conventional ureteric stent removal [17].

Capocasale et al. also used the VAS to assess pain on the removal of the ureteric stents [15]. While no numerical value was given, this article reported that 93 patients described discomfort while 7 patients described distress on removal [15]. However, the removal of magnetic ureteric stents was performed in a clinic for all patients except 7, who were still inpatients due to medical issues [15]. Flexible cystoscopy was required for magnetic ureteric stent removal for 2 patients’ magnetic stent group in this study, 1 due to encrustation of the stent and the second due to severely enlarged prostate gland [15]. This is similar to Rassweiler et al.*,* who also reported a 2% failure of removal via the magnet retriever, although they did not specify the reason for failure [14]. Sevcenco et al. had one patient who underwent laparoscopic pyeloplasty requiring removal of magnetic ureteric stent via a cystoscope due to encrustation [17]. O’Kelly et al. reported five patients who attended an emergency department with pain, following removal of the magnetic ureteric stent. These patients had their stent in situ for only 3–5 days, suggesting ureteric oedema has not resolved by this time [16].

Pohlman et al. was a single-centre cohort study that consisted of 7 kidney transplant recipients [18]. The purpose of the study was to assess the functional efficiency and practicality of the use of magnetic ureteric stents in kidney transplant patients. This study used the USSQ and VAS scoring to determine the quality of life and pain of the recipients, respectively. This study also recorded the cost reduction. All magnetic ureteric stents were successfully removed with a mean time of 3.4 min to remove the stents. The mean resulting pain experienced was 2.67 as per the VAS scoring system.

Furthermore, 2 out of the 6 patients did not experience any pain, and 2 patients experienced pain in the suprapubic region. One patient experienced pain in the flank region. The VAS score for the pain on the removal of the stent was measured at 2.6.

O’Connell et al. performed a retrospective cohort study of a single institute experience of the magnetic ureteric stents [19]. There were 59 participants, all of whom had treatment for urinary calculi. The authors used USSQ to assess the indwelling stent symptoms, with a response from 68% of the participants. The incidence of stent discomfort was 69%. Although 25% of the participants are reported as experiencing debilitating pain secondary to the indwelling stents, only 30% of the participants described stent-related symptoms as bothersome [19]. There were no difficulties experienced during the removal of the magnetic ureteric stents and no failed retrievals of the stents [19].

### Secondary outcomes

The secondary outcome of this review is to assess the cost-effectiveness of using magnetic vs. conventional ureteric stent. All six studies showed a reduction in cost using magnetic ureteric stents. This is most likely due to the inclusion of sterilisation costs required per cystoscopically removed conventional ureteric stents [14]. The cost per patient was calculated by O’Kelly et al. and Sevcenco et al. *studies*, showing a saving per patient €203.00 [16, 17]. O’Connell et al. calculated the cost-saving for the 9-month period of their study, which accounted for €47,790 [19].

### Assessment of bias

Utilising the Newcastle Ottawa Scale, the risk of bias was medium for the three comparative cohort studies (Table 2). Similarly, the modified Delphi criteria suggested the two non-comparative case series were of low quality (Table 3). None of these studies described conflicts of interest or funding.

**Table 2:** Risk of bias amongst comparative cohort studies, per the Newcastle–Ottawa Quality Assessment Scale

Year	First author	1. Selection				2. Comparability	3. Outcome			Total
		Representativeness of exposed cohort	Selection non-exposed cohort	Ascertainment of exposure	Demonstration outcomes not present at start of study	Comparability of cohorts	Assessment of outcome	Was follow-up long enough for outcomes to occur?	Adequacy of follow-up of cohorts	
2017	Sevcenco	*	*	*	n/a	*	*	n/a	n/a	5
2019	O’Kelly	*	*	*	n/a	*	*	n/a	n/a	5
2019	Capocasale	*	*	*	n/a	*	*	n/a	n/a	5

Studies scoring 0–2, 3–4 and 5–6 points were identified as high, medium and low risk of bias, respectively

*n*/*a* not required

^*^ Indicates one point. ** Indicates two points

**Table 3:** Assessing risk of bias amongst non-comparative cases series. Modified Delphi criteria checklist

Year	First author	Study objective	Study population					Intervention (s)		Outcome measure			Statistical analysis	Results and conclusions					Competing interests	Total score
		Cr 1	Cr 2	Cr 3	Cr 4	Cr 5	Cr 6	Cr 7	Cr 8	Cr 9	Cr 10	Cr 11	Cr 12	Cr 13	Cr 14	Cr 15	Cr 16	Cr 17	Cr 18	
2019	Pohlman et al.	*	*	*	-	-	-	*	-	*	-	-	-	n/a	-	-	-	-	-	4
2019	O'Connell et al.	*	*	-	-	-	-	*	-	*	-	-	-	n/a	-	-	-	-	-	3

Studies scoring 0–5, 6–10 and 11–15 points were identified as high, medium and low quality, respectively

*Cr* criterion, *n*/*a* not applicable

∗ Indicates one point

## Discussion

Ureteric stents are a key part of urological surgery, and their use is endorsed by several international urological associations [20]. Ureteric stents are, however, associated with significant morbidity, both while in situ and upon removal. The conventional ureteric stent, which is used worldwide, requires removal by flexible cystoscopy with a stent grasping forceps unless the strings are used for removal. This procedure is uncomfortable and costly, requiring trained staff, a procedure room, equipment, sterilisation and occasional repairs [21].

In this review, we analysed all studies comparing magnetic vs. conventional ureteric stents. We noted that magnetic stents were associated with reduced pain at the time of removal. Magnetic stents were not associated with fewer symptoms than conventional ureteric stents, as expected. However, only one study reported this when they examined self-reported symptoms over a 4-week period. But this could be interpreted as patients’ getting used to conventional ureteric stents and no longer finding them bothersome. There is no internationally accepted consensus on the ideal duration for ureteric stents to be left in situ. Results from O’Kelly et al. would suggest caution when considering early removal of ureteric stents, as a small number of patients presented to the emergency department with pain [16]. However, the authors did not report whether any patient required re-insertion of their ureteric stent. Two studies found magnetic ureteric stents were found to be viable options for ureteric stenting for kidney transplant procedures to avoid post-operative complications, and this finding was not an aim of this study but an interesting one nonetheless [15, 18]. They both discussed the benefit of reduction in pain with the removal of magnetic ureteric stents.

All the six studies reported that the removal of magnetic stents was more cost-effective than conventional ureteric stents. This is most likely due to the inclusion of sterilisation costs required per cystoscopically removed conventional ureteric stents [14]. The amount saved per stent removal procedure was approximately equivalent, ranging from €100 to €203. O’Connell et al. showed that not only could this mechanism of ureteric stenting reduce cost but can also reduce resources being utilised for stent removal, with a nurse run clinic able to perform magnetic stent removals [19]. This main limitation of this review was the low quality and size of identified studies. In addition, their methodological heterogeneity prohibited the performance of meta-analysis.

Three studies encountered issues with the retrieval of the magnet ureteric stent. Rassweiler et al., and Capocasale et al., both had a 2% failure of removal requiring flexible cystoscopy for removal of the stent [14, 15]. Capocasale et al., however, explains the reason for failure of magnetic ureteric stent removal; one stent was encrusted, and the second was due to a severely enlarged prostate [15]. Sevcenco et al. reported one incidence of failure of removal of magnetic stent with retriever. Interestingly, this patient had undergone a laparoscopic pyeloplasty, with a minimum stent duration of 4 weeks [17]. This implies that magnetic ureteric stents may not be the most appropriate ureteric stents for medium or long-term stenting.

## Conclusion

Low-level evidence suggests that indwelling magnetic ureteric stents have similar morbidity to conventional ureteric stents. However, magnetic ureteric stents are associated with less pain and discomfort at the time of removal when compared to cystoscopic removal of conventional ureteric stents. Magnetic ureteric stents are associated with a considerable cost saving when compared to conventional ureteric stents.

## Data Availability

Available on request.
